# Assessment of the potential of the MET inhibitor tepotinib to affect the pharmacokinetics of CYP3A4 and P-gp substrates

**DOI:** 10.1007/s10637-023-01378-z

**Published:** 2023-07-06

**Authors:** Özkan Yalkinoglu, Andreas Becker, Axel Krebs-Brown, Claudia Vetter, Christian Lüpfert, Dominique Perrin, Jürgen Heuer, Herlind Biedert, Stefan Hirt, Afrim Bytyqi, Angelika Bachmann, Rainer Strotmann

**Affiliations:** 1 Clinical Pharmacology, Quantitative Pharmacology, the healthcare business of Merck KGaA, Darmstadt, Germany; 2 Global Biostatistics, Epidemiology and Medical Writing, the healthcare business of Merck KGaA, Darmstadt, Germany; 3 NCE DMPK, Discovery and Development Technologies, the healthcare business of Merck KGaA, Darmstadt, Germany; 4 Clinical Services, Nuvisan GmbH, Neu-Ulm, Germany; 5 LC/MS Bioanalysis, Nuvisan GmbH, Neu-Ulm, Germany

**Keywords:** Tepotinib, Drug-drug interactions, CYP3A4, P-glycoprotein, MET inhibitor

## Abstract

Tepotinib is a highly selective, potent, mesenchymal-epithelial transition factor (MET) inhibitor, approved for the treatment of non-small cell lung cancer harboring *MET* exon 14 skipping alterations. The aims of this work were to investigate the potential for drug-drug interactions via cytochrome P450 (CYP) 3A4/5 or P-glycoprotein (P-gp) inhibition. In vitro studies were conducted in human liver microsomes, human hepatocyte cultures and Caco-2 cell monolayers to investigate whether tepotinib or its major metabolite (MSC2571109A) inhibited or induced CYP3A4/5 or inhibited P-gp. Two clinical studies were conducted to investigate the effect of multiple dose tepotinib (500 mg once daily orally) on the single dose pharmacokinetics of a sensitive CYP3A4 substrate (midazolam 7.5 mg orally) and a P-gp substrate (dabigatran etexilate 75 mg orally) in healthy participants. Tepotinib and MSC2571109A showed little evidence of direct or time-dependent CYP3A4/5 inhibition (IC_50_ > 15 μM) in vitro, although MSC2571109A did show mechanism-based CYP3A4/5 inhibition. Tepotinib did not induce CYP3A4/5 activity in vitro, although both tepotinib and MSC2571109A increased CYP3A4 mRNA. In clinical studies, tepotinib had no effect on the pharmacokinetics of midazolam or its metabolite 1’-hydroxymidazolam. Tepotinib increased dabigatran maximum concentration and area under the curve extrapolated to infinity by 38% and 51%, respectively. These changes were not considered to be clinically relevant. Tepotinib was considered safe and well tolerated in both studies. The potential of tepotinib to cause clinically relevant DDI with CYP3A4- or P-gp-dependent drugs at the clinical dose is considered low. Study 1 (midazolam): NCT03628339 (registered 14 August 2018). Study 2 (dabigatran): NCT03492437 (registered 10 April 2018).

**Supplementary Information:**

The online version contains supplementary material available at 10.1007/s10637-023-01378-z.

## Introduction

Tepotinib is a once-daily, highly selective, potent, oral, reversible, adenosine triphosphate competitive, small molecule mesenchymal-epithelial transition factor (MET) inhibitor [1]. Following its first approval in Japan in March 2020 for treatment of advanced NSCLC harboring *MET* exon 14 (*MET*ex14) skipping, tepotinib has been approved by multiple regulatory authorities worldwide [2–6]. Guidelines now recommend tepotinib for advanced NSCLC harboring *MET*ex14 skipping, regardless of lines of prior therapy [7–9].

Tepotinib inhibits HGF-dependent and -independent MET phosphorylation and showed antitumor activity in multiple preclinical tumor models derived from diverse cancer types. The antitumor activity of tepotinib was particularly pronounced in tumors with oncogenic alterations of *MET*, such as *MET*ex14 skipping and high-level *MET* amplification [10, 11]. In clinical studies, tepotinib 500 mg once daily (QD) has demonstrated meaningful efficacy in an ongoing Phase 2 study of tepotinib in patients with NSCLC and confirmed *MET*ex14 skipping (VISION, NCT02864992) [12]. The dose selection was supported by translational pharmacokinetic-pharmacodynamic modeling of preclinical and clinical data [13].

For all new medicines, and particularly those used in indications likely to require polypharmacy, such as oncology, it is important to assess the potential for drug-drug interactions (DDIs). An important focus during drug development is the potential for drugs to modulate the activity of enzymes and transporters that may affect the pharmacokinetics of co-administered drugs. Cytochrome P450 (CYP) enzymes are an important group of enzymes and in particular CYP3A4, which is a major enzyme involved in the oxidative metabolism of many drugs [14, 15]. In addition, drug transporters such as P-glycoprotein (P-gp) often play a key role in the absorption and excretion of drugs, and are also implicated in multi-drug resistance [16].

The metabolic fate of tepotinib has been investigated in a human mass balance study [17], and it was found to be extensively cleared by the liver. Tepotinib is cleared as unchanged drug via biliary excretion and is also extensively metabolized. The majority of the total circulating radioactivity in plasma was found to comprise of tepotinib (55%) and its major circulating metabolite M506 (41%). M506 is a chiral mixture; the R-enantiomer MSC2571109A accounted for 64.6% and the S-enantiomer MSC2571107A accounted for only about 4.5% of the exposure of the parent drug. Despite its relevant plasma exposure, M506 is not a relevant elimination pathway with only minor amounts found in excreta [17].

This article presents the in vitro studies used to evaluate the risks of tepotinib and its metabolites to perpetrate DDIs via CYP3A4 and P-gp mediated mechanisms, and the clinical data used to assess the effect of tepotinib, at a therapeutic dose, on the pharmacokinetics of sensitive index CYP3A4 and P-gp substrates (midazolam and dabigatran, respectively).

## Methods

### In vitro studies

#### Investigation of CYP450 inhibition and induction

The potential of tepotinib and MSC2571109A to inhibit CYP450 enzymes CYP1A2, 2A6, 2B6, 2C8, 2C9, 2C19, 2D6, 2E1, and 3A4/5 in vitro was evaluated using CYP-catalyzed reactions in pooled mixed-gender human liver microsomes using specific substrates and liquid chromatography/tandem mass spectrometry (LC–MS/MS) readouts. Notably, the CYP3A4/5 catalyzed reactions used in the tepotinib studies included midazolam 1-hydroxlation, testosterone 6β-hydroxylation and nifedipine oxidation. Testosterone 6β-hydroxylation was assessed in the MSC2571109A study. Assessments of mechanism-based inhibition of the above listed CYP450 enzymes were conducted after pre-incubation of the microsomes with tepotinib or MSC2571109A. A brief summary of the incubation methods, including details of substrates used for the other CYP enzymes (which are not pertinent to this article) are presented in Online Resource 1. The LC–MS/MS-based methods used were consistent with those previously described in the literature [18–21].

In vitro studies in primary human hepatocyte cultures were conducted to assess the potential of tepotinib to induce human CYP1A1/2, 2B6 and 3A4/5 activity, the latter based on testosterone 6β-hydroxylation reactions (Online Resource 2). Further studies were conducted in human hepatocyte cultures to assess the potential of tepotinib and MSC2571109A to increase messenger ribonucleic acid (mRNA) levels of the above-mentioned CYPs (Online Resource 3) as this has been shown to be a more sensitive marker of induction than CYP3A4 activity [22]. The studies included positive (rifampin) and vehicle (DMSO) controls. A negative control (flumazenil) was also included in the mRNA studies. The study designs, test systems and selection of prototypical inducers used in these studies were consistent with PhRMA, FDA and EMA guidelines [20, 23–25].

The risks of clinically significant CYP-mediated perpetrator DDIs were assessed for tepotinib and MSC2571109A, at the plasma levels expected for the proposed clinical dose of 500 mg tepotinib, using static modeling [26]. MSC2571107A was not investigated as an inhibitor or inducer of CYP3A4 given it is a minor metabolite.

#### Investigation of P-gp inhibition

An in vitro assay in Caco-2 cell monolayers was performed to assess the P-gp inhibition potential of tepotinib (Online Resource 4). [^3^H]-digoxin was used as a P-gp probe substrate. Control experiments were conducted to ensure acceptable performance of the cells. The extent of inhibition in these studies, expressed as IC_50_, was determined by quantification of the transport or uptake of ^3^H-digoxin in Caco-2 cells in the absence and presence of tepotinib.

To assess the potential clinical risk and to identify whether an in vivo DDI study was required, drug concentration ([I])/IC_50_ ratios and/or R-values were calculated considering the mean human steady state exposure of parent drug and its metabolite, observed at the clinical dose of 500 mg tepotinib. Results were assessed according to both FDA and EMA criteria [20, 24].

### Clinical studies to investigate DDIs with CYP3A4 or P-gp substrates

#### Study design

Two clinical studies were conducted to assess the potential of tepotinib to affect the pharmacokinetics of sensitive CYP3A4 and P-gp index substrates, midazolam and dabigatran etexilate. Safety and tolerability were also assessed throughout each study.

**Fig. 1:**
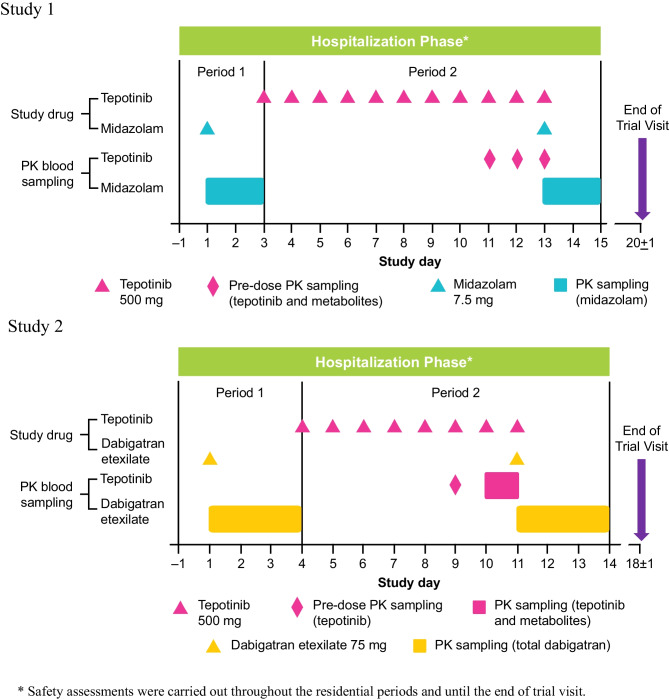
Study design figures

Study 1 (NCT03628339) and Study 2 (NCT03492437) were both Phase 1, open-label, single sequence, 2-period studies in 12 and 20 healthy participants, respectively (Fig. 1).

In Study 1, Period 1, participants received a single oral dose of 7.5 mg midazolam on Day 1, 4 h after completion of a standardized continental breakfast. In Period 2 (Days 3 to 15), participants received 500 mg tepotinib, QD 30 min after starting a continental breakfast on Days 3 to 12. On Day 13, participants received the final dose of 500 mg tepotinib 30 min after starting a standardized breakfast identical to the one on Day 1. A single oral dose of midazolam 7.5 mg was administered 4 h after tepotinib administration on Day 13. Blood samples for midazolam and 1-hydroxymidazolam plasma concentrations were collected from predose to 43 h after midazolam administration on Days 1 and 13. Blood samples for trough concentrations of tepotinib were collected on Days 11, 12 and 13.

In Study 2, Period 1, participants received a single oral dose of 75 mg dabigatran etexilate on Day 1. In Period 2 (Days 4 to 14), participants received 500 mg tepotinib QD on Days 4 to 11. In addition, on Day 11, participants received a single oral dose of 75 mg dabigatran etexilate taken at the same time as the tepotinib dose. All doses of tepotinib and dabigatran etexilate were taken 30 min after starting a standardized breakfast. Blood samples for total dabigatran (unconjugated plus conjugated) plasma concentrations were collected from predose to 72 h after dabigatran etexilate administration on Days 1 and 11. Blood samples for trough concentrations of tepotinib were collected on Days 9 and 10. Further blood samples for tepotinib and its metabolites were also collected up to 24 h postdose on Day 10.

For both studies, participants were screened up to 28 days prior to the first dose. Enrolled participants were resident from the day prior to dosing in Period 1 (Day -1), through to completion of assessments in Period 2 (i.e., Day 15 in Study 1, and Day 14 in Study 2). An End of Study visit was conducted 7 days (± 1 day) after the last dose in both studies.

#### Participants

All participants had to provide written informed consent prior to participation. Eligible participants were healthy males or females, aged 18 to 44 years, with a body mass index of ≥ 18.5 and ≤ 29.9 kg/m^2^ and a body weight of 50 to 100 kg. Female participants had to be of nonchildbearing potential. Male participants had to use a barrier method and have their female partner of childbearing potential use highly effective methods of contraception throughout the studies and for at least 3 months after the last study drug administration.

Exclusion criteria included evidence or history of clinically significant conditions that in the opinion of the investigator constituted a risk or contraindication for participation, or that could interfere with study objectives. Use of any prescription or nonprescription medications (except for paracetamol) within 14 days or 5 half-lives of Day 1, and participation in the treatment phase of a clinical study within 60 days or 5 half-lives before Day 1 was not permitted. Intake of grapefruit, Seville orange, cranberry or juices of these fruits, or St John’s Wort was not permitted for 14 days prior to Day -1. In Study 2, participants with an increased risk of bleeding were also excluded.

#### Pharmacokinetic analysis

Plasma concentrations of midazolam and 1-hydroxymidazolam, total dabigatran, tepotinib and its metabolites were measured using validated liquid chromatography and tandem mass spectrometry methods as previously described [17].

Pharmacokinetic parameters were calculated using standard noncompartmental methods using the software Phoenix WinNonlin (Certara, L.P., Princeton, New Jersey, version 6.2 or higher). Parameters included maximum plasma concentration (C_max_) and time to reach C_max_ (T_max_) taken directly from the observed data. Area under the plasma concentration–time curve (AUC) from time 0 to the last measured concentration (AUC_0-t_), AUC over the dose interval (AUC_tau_) and AUC extrapolated to infinity (AUC_0-∞_) were calculated using the mixed log/linear trapezoidal rule. Terminal elimination half-life (t_1/2_) was calculated as ln(2)/λ_z_ where λ_z_ is the terminal rate constant. The primary pharmacokinetic parameters in both studies were C_max_, AUC_0-t_ and AUC_0-∞._

#### Statistical analysis and sample size determination

A similar statistical approach was taken for both studies. To estimate the magnitude of any effect of tepotinib on the index substrates (i.e., midazolam and total dabigatran) a linear model with Treatment and Participant as fixed effects was applied to log transformed pharmacokinetic parameters C_max_, AUC_0-t_ and AUC_0-∞_ of the substrates. Treatment differences on the log-scale of midazolam or dabigatran with tepotinib versus midazolam or dabigatran alone, respectively, were estimated for C_max_, AUC_0-t_ and AUC_0-∞_ together with their 90% confidence intervals (CI). Point estimates and CIs were back-transformed to the original scale.

For Study 1, assuming a coefficient of variation (CV) of 50% for midazolam AUC_0-t_, a one-sided alpha of 0.05, and a treatment ratio for the geometric means ranging between 0.85 and 1.18, the power to exclude a moderate effect (i.e., the 90% CI not falling into the [0.50 – 2.00] interval) was 80% with 12 evaluable subjects. A total of 12 participants were recruited.

For Study 2, assuming a CV of 30% for dabigatran AUC_0-t_, a one-sided alpha of 0.05 and a treatment ratio for the geometric means of less than 1.5, the power to exclude a moderate effect (i.e., the upper limit of the 90% CI exceeding 2.00) was 80% with 16 evaluable subjects. With a treatment ratio of less than 1.14, the power to exclude a mild effect (i.e., the upper limit of the 90% CI exceeding 1.5) was also 80% with 16 evaluable subjects. A total of 20 subjects were recruited to allow for drop-outs.

## Results

### In vitro studies

#### CYP inhibition and induction

Tepotinib and MSC2571109A were evaluated as potential inhibitors of human CYP enzymes CYP1A2, 2A6, 2B6, 2C8, 2C9, 2C19, 2D6, 2E1, and 3A4/5. Tepotinib inhibited CYP2C8-, CYP2C9-, CYP2C19- and CYP3A4/5-catalyzed probe substrate reactions in vitro in human liver microsomes with Ki values ≥ 11 µM whilst MSC2571109A was a direct inhibitor of CYP2C9 in vitro with a Ki value of 4.4 µM. CYP inhibition results for tepotinib and MSC2571109A are summarized in Table 1 and Online Resource 5. MSC2571109A was a mechanism-based inhibitor of CYP3A4/5 enzyme based on the increased inhibition observed at the highest tested concentration of 15 µM (data on file) after preincubation.

**Table 1:** Summary of in vitro evaluation of tepotinib as an inhibitor of human CYP enzymes

**Enzyme**	**CYP reaction**	**Direct inhibition**				**Time-dependent inhibition**		
		**Zero-minute preincubation**				**30-min**		**Potential for** **time-dependent** **inhibition**
		**IC**_**50**_** (µM)**	**Maximum** **inhibition at** **49.5 µM (%)**	**Ki (µM)**	**Type of** **inhibition**	**IC**_**50**_** (µM)**	**Maximum** **inhibition at** **49.5 µM (%)**	
CYP1A2	Phenacetin O-dealkylation	> 49.5	11	ND	ND	> 49.5	26	Little or No
CYP2A6	Coumarin 7-hydroxylation	> 49.5	51	ND	ND	39	52	Little or No
CYP2B6	Efavirenz 8-hydroxylation	> 49.5	34	ND	ND	44	52	Little or No
CYP2C8	Amodiaquine N-dealkylation	16	86	11	Mixed	13	86	Little or No
CYP2C9	Diclofenac 4-hydroxylation	27	58	ND	ND	23	52	Little or No
CYP2C19	S-Mephenytoin 4-hydroxylation	30	64	ND	ND	29	65	Little or No
CYP2D6	Dextromethorphan O-demethylation	> 49.5	50	ND	ND	> 49.5	24	Little or No
CYP2E1	Chlorzoxazone 6-hydroxylation	> 49.5	19	ND	ND	> 49.5	17	Little or No
CYP3A4/5	Testosterone 1’-hydroxylation	31	62	ND	ND	37	59	Little or No
CYP3A4/5	Midazolam 6β-hydroxylation	25	66	ND	ND	33	60	Little or No
CYP3A4/5	Nifedipine oxidation	26	64	ND	ND	25	71	Little or No

Tepotinib did not induce human CYP1A1/2, 2B6, and 3A4/5 activities to a relevant extent (less than 40% of positive control), as evaluated in primary human hepatocyte cultures based on enzyme activity quantification. However, tepotinib induced an up to 7-fold increase in CYP3A4 mRNA in a concentration-dependent manner in a separate study based on mRNA quantification whilst MSC2571109A induced up to 23-fold increase in CYP3A4 mRNA and up to 7-fold increase in CYP2B6 mRNA. Based on mechanistic static modeling, a theoretical potential risk for clinical DDIs was identified for CYP3A4/5 but could be excluded for other CYP isoforms.

#### In vitro transporter inhibition

Tepotinib was shown to inhibit P-gp with an apparent IC_50_ value of 0.41 μM. The calculated [I]/IC_50_ ratios based on the proposed clinical dose of 500 mg once daily were 8917 (intestinal) and 0.39 (systemic). Both ratios exceeded regulatory cutoff values [20, 24], suggesting a potential risk for clinically relevant interactions. As the parent compound was shown to inhibit P-gp, the effect of the metabolite MSC2571109A on P-gp was not investigated in vitro.

### Clinical results

#### Study population

In Study 1, a total of 12 male participants were treated of whom the majority (83%) were Caucasian and the mean age was 35 years (range 19 to 44 years). All 12 participants completed the study and all were included in the pharmacokinetic and safety analyses. The study was conducted in a single center between August and October 2018.

In Study 2, a total of 20 participants were treated of whom the majority were male (95%) and Caucasian (95%). The mean age was 33 years (range 20 to 43 years). One participant discontinued (withdrew consent) from the study on Day 10 prior to tepotinib dosing. All 20 participants were included in the pharmacokinetic and safety analyses. The study was conducted in a single center between May and August 2018.

#### Effect of tepotinib on CYP3A4

**Fig. 2:**
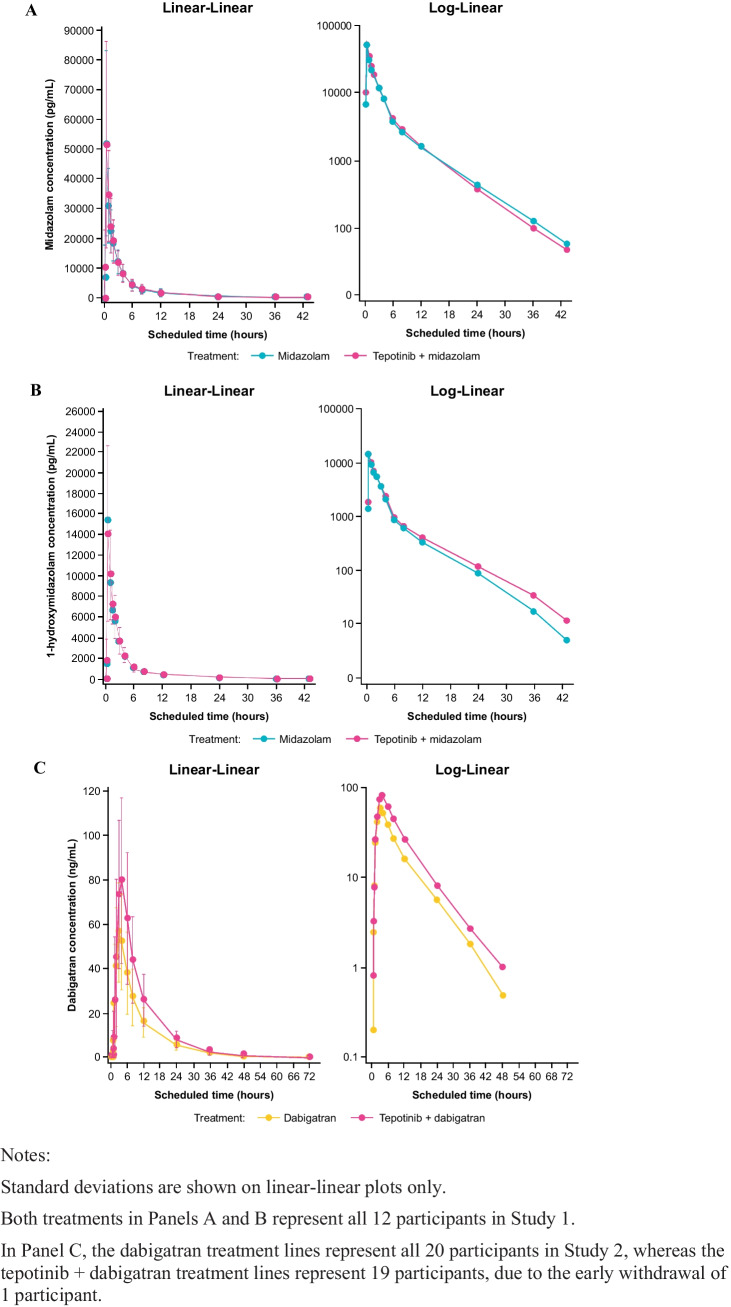
Mean (standard deviation) plasma concentration–time profiles for midazolam (**A**) and 1-hydroxymidazolam (**B**) following midazolam (7.5 mg single dose) administered alone and in the presence of tepotinib (500 mg once daily), and for dabigatran (**C**) following dabigatran etexilate (75 mg single dose) administered alone and in the presence of tepotinib (500 mg once daily)

Multiple dose treatment with 500 mg tepotinib QD did not affect the systemic exposure of midazolam or its main metabolite 1-hydroxymidazolam. Trough concentrations suggest tepotinib was at steady-state when midazolam was coadministered (Online Resource 6). Mean concentration–time profiles for both analytes following administration of midazolam in the presence and absence of tepotinib were almost identical (Fig. 2A, B). All pharmacokinetic parameters of midazolam and 1-hydroxymidazolam were similar following administration of midazolam in the presence and absence of tepotinib (Table 2).

**Table 2:** Summary of midazolam and 1-hydroxymidazolam pharmacokinetic parameters and statistical analysis of midazolam pharmacokinetic parameters

Parameter^a^	Midazolam (N = 12)	Tepotinib + Midazolam (N = 12)	Ratio of Geometric LS Mean (%) (90% CI)^b^
**Midazolam**			
AUC_0-t_ (h*pg/mL)	107,969 (42)	109,477 (47)	101.40 (89.35, 115.06)
AUC_0-∞_ (h*pg/mL)	109,285 (42)	110,550 (46)	101.16 (89.24, 114.66)
C_max_ (pg/mL)	49,172 (60)	50,954 (54)	103.62 (86.91, 123.56)
T_max_ (h)	0.500 (0.500, 1.50)	0.508 (0.483, 2.00)	NC
t_1/2_ (h)	5.52 (31)	4.81 (41)	NC
**1-hydroxymidazolam**			
AUC_0-t_ (h*pg/mL)	30,589 (24)	32,627 (25)	NC
AUC_0-∞_ (h*pg/mL)	31,269 (23)	33,372 (24)	NC
C_max_ (pg/mL)	14,909 (60)	15,852 (35)	NC
T_max_ (h)	0.500 (0.500, 2.00)	0.508 (0.483, 2.00)	NC
t_1/2_ (h)	6.34 (30)	6.90 (27)	NC
MR_AUC (ratio)	0.286 (37)	0.302 (34)	

AUC_0-t_ = area under the plasma concentration–time curve from time 0 to the last measurable concentration; AUC_0-∞ =_ area under the plasma concentration–time curve from time 0 extrapolated to infinity; C_max_ = maximum plasma concentration; LS = least squares; MR_AUC = metabolic ratio of 1-hydroxymidazolam AUC_0-∞_ and midazolam AUC_0-∞_; NC = not calculated; T_max_ = time to maximum concentration, t_1/2_ = terminal elimination half-life

^a^ Values are presented as geometric means (geometric CV%) for all parameters except T_max_ which is median (min, max)

^b^ Statistical comparisons were only conducted for the midazolam primary pharmacokinetic parameters

The estimates of the geometric least squares mean ratios (GMRs) for midazolam C_max_ and AUC parameters were approximately 100% (range 101.16 to 103.62) and the 90% CIs for the GMRs were within the accepted ‘no effect’ limits of 80 to 125%.

#### Effect of tepotinib on P-gp

Multiple dose treatment with 500 mg tepotinib QD mildly increased the systemic exposure of total dabigatran (Fig. 2C). Trough concentrations suggest tepotinib was at steady-state when dabigatran was coadministered (Online Resource 7). Mean dabigatran exposure parameters (C_max_ and AUC) were higher when dabigatran was coadministered with tepotinib (Table 3). The estimates of the GMRs for dabigatran C_max_, AUC_0-t_ and AUC_0-∞_ were approximately 138%, 151% and 145%, respectively. Dabigatran median T_max_ and mean t_1/2_ were similar for both treatments. A summary of the pharmacokinetic parameters for tepotinib and its metabolites is presented in Online Resource 8. There was no evidence of a relationship between tepotinib or MSC2571109A exposure and the relative bioavailability of dabigatran when administered with or without tepotinib (Online Resource 9 and Online Resource 10, respectively).

**Table 3:** Summary of dabigatran pharmacokinetic parameters and statistical analysis of dabigatran pharmacokinetic parameters

**Parameter**^**a**^	**Dabigatran** **(N = 20)**	**Tepotinib + Dabigatran** **(N = 19)**	**Ratio of Geometric** **LS Mean (%)** **(90% CI)**^**b**^
**Dabigatran**			
AUC_0-t_ (h*ng/mL)	461 (75)	709 (65)	151.38 (127.35, 179.93)
AUC_0-∞_ (h*ng/mL)	544 (33)^c^	730 (62)	144.70 (122.89, 170.39)
C_max_ (ng/mL)	54.5 (73)	76.9 (62)	138.45 (121.59, 157.65)
T_max_ (h)	3.00 (1.50, 6.00)	4.00 (2.00, 6.00)	NC
t_1/2_ (h)	8.18 (13)^c^	7.81 (14)	NC

AUC_0-t_ = area under the plasma concentration–time curve from time 0 to the last measurable concentration, AUC_0-∞ =_ area under the plasma concentration–time curve from time 0 extrapolated to infinity, C_max_ = maximum plasma concentration, LS = least squares, NC = not calculated, T_max_ = time to maximum concentration, t_1/2_ = terminal elimination half-life

^a^Values are presented as geometric means (geometric CV%) for all parameters except T_max_ which is median (min, max)

^b^Statistical comparisons were only conducted for the primary pharmacokinetic parameters

^c^N = 19

#### Safety

Treatments in both studies were considered safe and well tolerated (multiple dose dabigatran was considered to be reasonably well tolerated). There were no serious or severe adverse events, and no participants withdrew due to adverse events. Almost all adverse events were considered to be mild in severity.

The majority of adverse events were reported in Period 2 compared to Period 1 of both studies, with the majority of adverse events considered related to tepotinib rather than midazolam or dabigatran.

The most common adverse events in Study 1 (n = 12) were headache (5 participants), upper abdominal pain (4 participants) and diarrhea (4 participants). The most common adverse events in Study 2 (n = 20) were diarrhea (11 participants), upper abdominal pain (7 participants) and nausea (6 participants).

There were no clinically relevant changes in vital signs, electrocardiogram parameters or laboratory parameters in either study. Mild increases in serum creatinine were observed in some participants but all were transient, without clinical signs and returned to normal or nearly normal by the End of Treatment visit.

## Discussion

The intended target population of tepotinib is patients with advanced or metastatic NSCLC, who are mostly elderly and often receive multiple concomitant drugs including drugs that are metabolized by CYP3A4 or transported by P-pg. Therefore, characterization of the DDI perpetrator potential toward these targets is clinically relevant. Consistent with US and EU regulatory guidance [20, 24, 27], a systematic approach was adopted, whereby the potential for DDIs was first investigated in vitro, and only evaluated in clinical studies where in vitro findings suggested a relevant risk.

The potential for tepotinib and its major metabolite to induce or inhibit the major CYPs was investigated in vitro. The results of these studies suggested that tepotinib and MSC2571109A were weak direct inhibitors of CYP3A4/5 (IC_50_ values well above the geometric mean free C_max_ of 52.4 nmol/L [the healthcare business of Merck KGaA, Darmstadt, Germany, data on file]), and MSC2571109A showed mechanism-based inhibition of CYP3A4. In addition, both tepotinib and MSC2571109A increased CYP3A4 mRNA levels in vitro. In vitro studies suggested that tepotinib is also a P-gp inhibitor.

The results presented here are in general agreement with IC_50_ values reported in a recent study [28] with the exception of CYP2C9 and P-gp for which somewhat lower and higher values were reported, respectively. However, that study used generic substrates and a research-grade fluorometric method while here, specific substrates and LC–MS/MS methods were used.

Evaluation of the in vitro data against the FDA and EMA guidances, suggested that there was a need to conduct a clinical DDI study with sensitive substrates for both CYP3A4 and P-gp.

Midazolam is a short-acting sedative hypnotic benzodiazepine that is metabolized in the liver and gut wall by CYP3A4, and is established as a sensitive index substrate for CYP3A4 for clinical DDI studies [29]. Dabigatran etexilate is an anticoagulant prodrug and is recommended as a sensitive P-gp substrate for clinical DDI studies [29].

Based on the lack of genotoxic effects and the good overall safety profile of tepotinib, both of the clinical studies were conducted in healthy participants to minimize variability. The studies were designed to investigate the effect of steady-state tepotinib, at the clinical dose of 500 mg QD, on the pharmacokinetics of single oral doses of midazolam and dabigatran etexilate.

Midazolam absorption is delayed when administered in a nonfasted state, whereas tepotinib is recommended to be administered with food to improve its absorption. To minimize food effects on midazolam and to administer midazolam nearer to tepotinib C_max_, (median steady state tepotinib T_max_ = 8 h, with a range between 2 and 24 h), midazolam was administered 4 h after tepotinib.

Blood samples for midazolam and 1-hydroxymidazolam were collected over 43 h post-dose, which is > 5 times the terminal half-life of midazolam (~ 5 h) and therefore allowed adequate characterization of midazolam pharmacokinetic profile. Similarly, blood samples for dabigatran were collected over 72 h post-dose, which is > 5 times its terminal half-life (~ 8 h).

The results from Study 1 demonstrated that tepotinib at a therapeutic dose, did not affect the pharmacokinetics of either midazolam or its primary metabolite, suggesting that the potential for pharmacokinetic interactions with other CYP3A4 substrates is low.

The results from Study 2 suggested that tepotinib had a weak effect on the pharmacokinetics of the sensitive P-gp substrate dabigatran. Coadministration with tepotinib increased C_max_ and AUC of dabigatran compared to administration alone by approximately 40% and 50%, respectively. This is generally not considered clinically relevant, but the labeling information advises caution for P-gp substrates for which minimal concentration changes may lead to serious or life-threatening toxicities.

In summary, when tepotinib was administered orally at the therapeutic dose of 500 mg QD, there was no change in exposure to the sensitive CYP3A substrate, midazolam. There was less than a 2-fold exposure increase to dabigatran, indicating that tepotinib is a weak inhibitor of the P-gp-mediated efflux transport of the substrate dabigatran etexilate. Tepotinib was considered safe and well tolerated in these studies involving healthy participants. The potential of tepotinib to cause clinically relevant DDI with CYP3A4- or P-gp-dependent drugs at the intended posology is considered low.

## Supplementary Information

Below is the link to the electronic supplementary material.

Supplementary file1 (PPTX 50 KB)

Supplementary file2 (PPTX 213 KB)

## Data Availability

Any requests for data by qualified scientific and medical researchers for legitimate research purposes will be subject to the healthcare business of Merck KGaA, Darmstadt, Germany, Data Sharing Policy. All requests should be submitted in writing to the healthcare business of Merck KGaA, Darmstadt, Germany, data sharing portal, which can be found at https://www.emdgroup.com/en/research/our-approach-to-research-and-development/healthcare/clinical-trials/commitment-responsible-data-sharing.html. When the healthcare business of Merck KGaA, Darmstadt, Germany has a co-research, co-development, or co-marketing or co-promotional agreement, or when the product has been out-licensed, the responsibility for disclosure might be dependent on the agreement between parties. Under these circumstances, the healthcare business of Merck KGaA, Darmstadt, Germany, will endeavor to gain agreement to share data in response to requests.
