# Supplementary figures and images for: Assessment of the potential of the MET inhibitor tepotinib to affect the pharmacokinetics of CYP3A4 and P-gp substrates
Source: Invest New Drugs. 2023 Jul 6;41(4):596–605. doi: 10.1007/s10637-023-01378-z (PMC10447267; doi:10.1007/s10637-023-01378-z)

## Slide 1
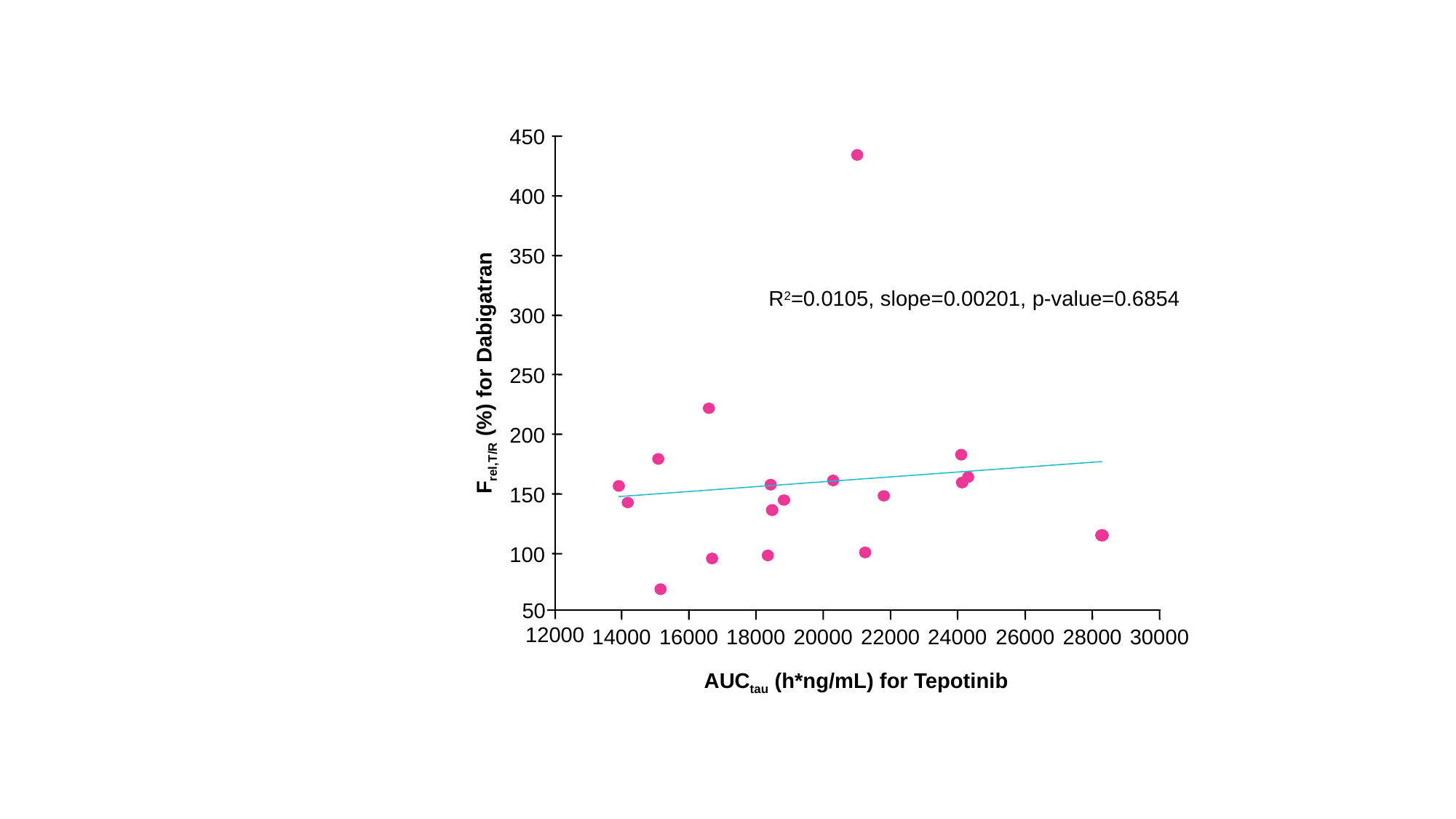

450
400
350
R2=0.0105, slope=0.00201, p-value=0.6854
300
250
Frel,T/R (%) for Dabigatran
200
150
100
50
12000
14000
16000
18000
20000
22000
24000
26000
28000
30000
AUCtau (h*ng/mL) for Tepotinib

Supplement: Supplementary file 1 — Supplementary file1 (PPTX 50 KB) [file 10637_2023_1378_MOESM1_ESM.pptx]
