# Supplementary material for: Assessment of the potential of the MET inhibitor tepotinib to affect the pharmacokinetics of CYP3A4 and P-gp substrates
Source: Invest New Drugs. 2023 Jul 6;41(4):596–605. doi: 10.1007/s10637-023-01378-z (PMC10447267; doi:10.1007/s10637-023-01378-z)
Supplement: Supplementary file 2 — Supplementary file2 (PPTX 213 KB) [file 10637_2023_1378_MOESM2_ESM.pptx]

## Slide 1
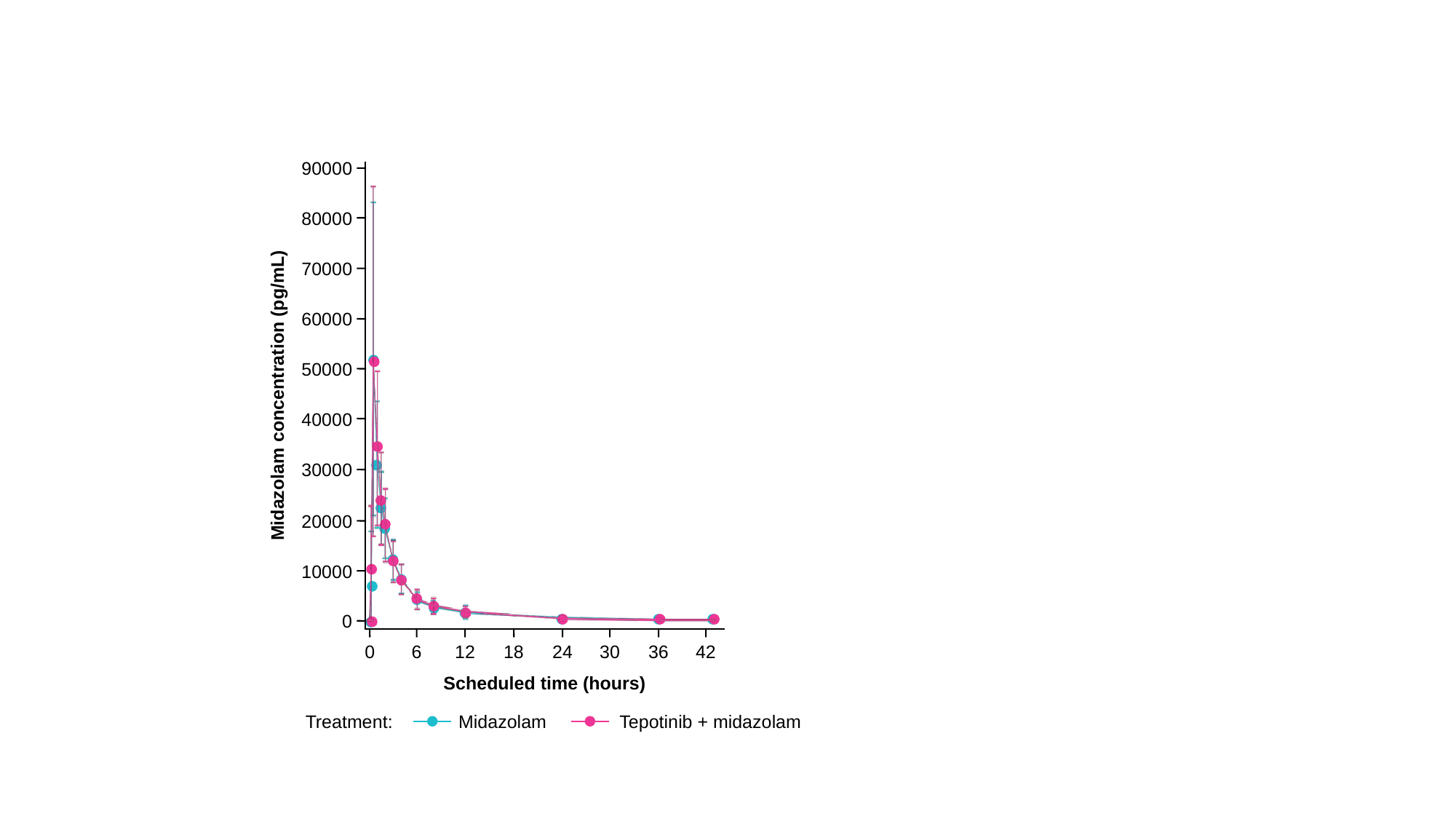

90000
80000
70000
60000
50000
Midazolam concentration (pg/mL)
40000
30000
20000
10000
0
0
6
12
18
24
30
36
42
Scheduled time (hours)
Treatment:
Midazolam
Tepotinib + midazolam
